# Nearly Perfect Transmission of Lamé Modes in a Rectangular Beam with Part and Through-Thickness Vertical Cracks

**DOI:** 10.3390/ma16114164

**Published:** 2023-06-02

**Authors:** Xuwei Cao, Jing Ni, Chun Shao, Xiao Yang, Chenggan Lou

**Affiliations:** 1 School of Mechanical Engineering, Hangzhou Dianzi University, Hangzhou 310018, China; nj2000@hdu.edu.cn (J.N.); shaochun@hdu.edu.cn (C.S.); yangxiao0431@foxmail.com (X.Y.); 2 Hangzhou Applied Acoustics Research Institute, Hangzhou 310023, China; lchg-715@sohu.com

**Keywords:** guided wave, Lamé mode, rectangular beam, crack, nearly perfect transmission

## Abstract

The guided waves in the uniform waveguide of rectangular cross-section exhibit complicated propagation and scattering characteristics due to the diversity of vibration modes. This paper focuses on the mode conversion of the lowest Lamé mode at a part-through or through-thickness crack. Firstly, the Floquet periodicity boundary condition is applied to derive the dispersion curves in the rectangular beam, which relates the axial wavenumber to the frequency. On this basis, the frequency domain analysis is conducted to investigate the interaction between the fundamental longitudinal mode in the vicinity of the first Lamé frequency and a part-through or through-thickness vertical or inclined crack. Finally, the nearly perfect transmission frequency is evaluated by extracting displacement and stress harmonic fields throughout the cross-section. It is shown that this frequency originates from the first Lamé frequency, increases with the crack depth, and decreases with the crack width. Between them, the crack depth plays a major role in the frequency variation. In addition, the nearly perfect transmission frequency is negligibly affected by the beam thickness, and such a phenomenon is not observed for inclined cracks. The nearly perfect transmission may have potential applications in the quantitative evaluation of crack size.

## 1. Introduction

The beam structures subjected to complex loadings may initiate cracks, resulting in a significant reduction in their service life [1,2,3,4]. Ultrasonic-guided waves have proven to be an efficient tool in nondestructive evaluation and structural health monitoring, because they can propagate over long distances within the waveguide, and their scattering behavior highly depends on the location and severity of damage [5,6,7,8,9].

Compared with the guided waves in an infinite plate, an additional pair of surfaces in a rectangular beam introduces more boundary reflections of coupled longitudinal and shear waves, making the formation of guided modes more complicated. The crosswise superposition method [10], collocation method [11], approximate theory [12], finite element method [13], and spectral finite element method [14,15,16] have been applied to study the propagation of guided waves, though not all of them work well for all types of modes in a rectangular beam with arbitrary aspect ratios over a wide frequency range.

On the other hand, a clear view of the interaction between guided waves and various types of damage is also crucial for damage detection in beam structures. Sun et al. [17] identified the size of the damage in a thick steel beam using the time-of-flight of the transmitted wave packet. Rucka [18] investigated the propagation of longitudinal and flexural waves in an intact bar, as well as in bars with an additional mass, a notch, and a grooved weld. Atashipour et al. [19] used damage characteristic points together with a multilayer feedforward artificial neural network supervised by an error-backpropagation algorithm to identify damage in thick steel beams. Hosseinabadi et al. [20] extracted three damage-sensitive features from guided wave signals, then input them to the established multiple-input multiple-output fixed grid wavelet network for estimating damage location and severity in a structural beam. Xu et al. [21] determined the depth of a partial-thickness notch by the mode-converted energy rate based on the finite difference time domain simulation. Ng et al. [22,23] and Wang [24] utilized the Bayesian approach to identify the crack parameters and the associated uncertainties by minimizing the discrepancy between the simulated data of the crack model and the measured data. Serey et al. [25] selectively generated an antisymmetric mode and inferred the position of a simulated defect in a rectangular aluminum bar. Cheng et al. [26] located the damage/crack in an I-shaped steel girder based on guided wave simulation and artificial neural networks. Most of the aforementioned techniques realized damage detection by extracting the time-of-flight or amplitude/energy of scattered modes in the beam.

Lamé modes [27,28,29] and Mindlin–Fox modes [30] are two special classes of exact solutions that can only be given at certain frequencies and/or aspect ratios. In 1852, Lamé [27] constructed exact analytical solutions for a rectangular waveguide of longitudinal and bending types of free vibrations. These solutions solely comprised of shear waves are specifically known as Lamé equivoluminal modes, which occur at a series of discrete frequencies for arbitrary aspect ratios. Li et al. [31] applied the Fourier-phase method to measure the dispersion branches of S0 and SH1 plate modes in the vicinity of the first Lamé frequency in polycrystalline aluminum plates with weak anisotropy. Then a quantitative formula was presented to infer the texture parameters based on the amount of crossover or splitting of these two dispersion branches. Cao et al. [32] employed the mode matching method to investigate the interaction of S0 plate mode with a shallow crack of various depths. It was found that the frequency of the non-mode-conversion point originates from the first Lamé frequency and increases linearly with the crack depth. To the best of our knowledge, rare literature focuses on the damage evaluation in beam structures using Lamé modes.

The nearly perfect transmission phenomenon for electromagnetic and acoustic waves has been extensively researched [33,34,35,36,37,38], while analogous studies have seldom been reported in the field of ultrasonic guided wave based damage detection. In this paper, the interaction between the first Lamé mode and a part-through or through-thickness crack of various sizes is analyzed quantitatively. Based on the normal mode theory for beam structures of rectangular cross-section, the nearly perfect transmission may be observed when the crack size and the harmonic frequency satisfy a specific relation. The rest of this paper is structured as follows. Section 2 introduces the basic theory of normal guided modes in rectangular beams, especially the Lamé modes. Section 3 presents the method to search for the nearly perfect transmission frequency based on our defined scattering conversion ratios. Section 4 discusses the effects of crack width, axial extent and beam thickness on the nearly perfect transmission frequency. Section 5 draws the conclusions.

## 2. Guided Wave Modes in Beams

### 2.1. Normal Mode Theory

**Figure 1:**
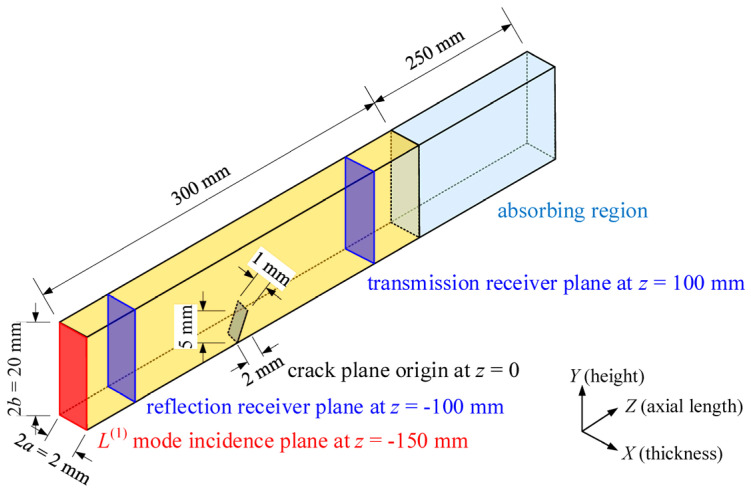
Example for guided waves propagating in a rectangular beam with a part-through-thickness inclined crack (not to scale).

Time-harmonic guided waves propagate along the axial direction (+*z*) of an isotropic elastic beam of rectangular cross-section (see Figure 1). With the help of Floquet periodicity boundary conditions in COMSOL Multiphysics 5.6 [13,39], the guided wave dispersion curves in an aluminum beam (Young’s modulus 69 GPa, Poisson’s ratio 0.33, density 2700 kg/m^3^, longitudinal wave velocity *c_L_* = 6150 m/s, shear wave velocity *c_T_* = 3100 m/s, thickness 2*a* = 2 mm, height 2*b* = 20 mm) are obtained.

**Figure 2:**
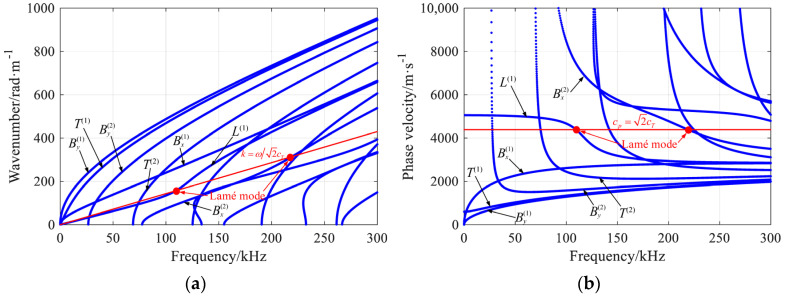
The (**a**) wavenumber dispersion curves and (**b**) phase velocity dispersion curves of guided waves in the rectangular beam in Figure 1.

For any two solutions “1” and “2” in elastic waveguides without external forces, the complex reciprocity relation [28] relates the velocity vector **v** to the stress tensor **T** by

(1) $$\nabla \cdot (\cdots )=\frac{\partial }{\partial x}(\cdots )\cdot \hat{x}+\frac{\partial }{\partial y}(\cdots )\cdot \hat{y}+\frac{\partial }{\partial z}(\cdots )\cdot \hat{z}=0$$

with

$$(\cdots )=\left({v_{2}}^{*}\cdot T_{1}+v_{1}\cdot {T_{2}}^{*}\right)$$

where ▽ depicts the divergence operator, ∂ denotes the partial derivative, the superscript * represents the complex conjugate, $\hat{x}$, $\hat{y}$, $\hat{z}$ are unit vectors in the *x*, *y*, *z* directions, respectively. The velocity and stress fields resulting from two modes with axial wavenumbers *k_m_*, *k_n_* at angular frequency *ω* can be expressed by their velocity/stress mode shapes (independent of *z*):

(2) $$\begin{array}{l} v_{1}=v_{m}(x,y)e^{i\left(k_{m}z-\omega t\right)},T_{1}=T_{m}(x,y)e^{i\left(k_{m}z-\omega t\right)}\\ v_{2}=v_{n}(x,y)e^{i\left(k_{n}z-\omega t\right)},T_{2}=T_{n}(x,y)e^{i\left(k_{n}z-\omega t\right)} \end{array}$$

Substituting Equation (2) into the complex reciprocity relation Equation (1), i.e.,

(3) $$\begin{array}{l} \;-i\left(k_{m}-{k_{n}}^{*}\right)\left({v_{n}}^{*}\cdot T_{m}+v_{m}\cdot {T_{n}}^{*}\right)\cdot \hat{z}\\ =\frac{\partial }{\partial x}\left({v_{n}}^{*}\cdot T_{m}+v_{m}\cdot {T_{n}}^{*}\right)\cdot \hat{x}+\frac{\partial }{\partial y}\left({v_{n}}^{*}\cdot T_{m}+v_{m}\cdot {T_{n}}^{*}\right)\cdot \hat{y} \end{array}$$

Performing the integral over the entire beam cross section, from *x* = −*a* to *x* = *a*, from *y* = −*b* to *y* = *b*, it follows that

(4) $$\begin{array}{l} \;-i\left(k_{m}-{k_{n}}^{*}\right)\int_{-b}^{b}\int_{-a}^{a}\left({v_{n}}^{*}\cdot T_{m}+v_{m}\cdot {T_{n}}^{*}\right)\cdot \hat{z}dx\;dy\\ =\int_{-b}^{b}\left[\left({v_{n}}^{*}\cdot T_{m}+v_{m}\cdot {T_{n}}^{*}\right)\cdot {\hat{x}|}_{x=-a}^{x=a}\right]dy+\int_{-a}^{a}\left[{\left({v_{n}}^{*}\cdot T_{m}+v_{m}\cdot {T_{n}}^{*}\right)\cdot \hat{y}|}_{y=-b}^{y=b}\right]dx \end{array}$$

The stress-free boundary conditions for beam surfaces yield ${T\cdot \hat{x}|}_{x=\pm a}={T\cdot \hat{y}|}_{y=\pm b}$ = **0**, and RHS of the above equation is equal to zero. As all field quantities vary as $e^{-i\omega t}$, the velocity mode shape **v** in Equation (4) is converted to the displacement mode shape **u**, that is

(5) $$i\left(k_{m}-{k_{n}}^{*}\right)4P_{mn}=0$$

where

(6) $$\begin{array}{ll} P_{mn} & =-\frac{1}{4}\int_{-b}^{b}\int_{-a}^{a}\left({v_{n}}^{*}\cdot T_{m}+v_{m}\cdot {T_{n}}^{*}\right)\cdot \hat{z}dx\;dy\\ & =-\frac{i\omega }{4}\int_{-b}^{b}\int_{-a}^{a}\left({u_{n}}^{*}\cdot T_{m}-u_{m}\cdot {T_{n}}^{*}\right)\cdot \hat{z}dx\;dy\\ & =-\frac{i\omega }{4}\int_{-b}^{b}\int_{-a}^{a}\sum_{j=x,y,z}\left(u_{j,n}^{*}\cdot \sigma_{jz,m}-u_{j,m}\cdot \sigma_{jz,n}^{*}\right)dx\;dy \end{array}$$

Equation (5) indicates that *P_mn_* = 0 unless the axial wavenumbers of two modes satisfy the complex conjugate condition, i.e., $k_{m}=k_{n}^{*}$. This establishes the orthogonality relation for normal modes. On the other hand, *P_mm_* represents the axial power flow of a propagating mode with real wavenumber *k_m_* passing through the cross section. By normalizing the axial power flow of each mode, the scattering coefficient straightly corresponds to the respective magnitude, thus simplifying the calculation in Section 3.

### 2.2. Lamé Modes in Rectangular Beams

According to the twofold symmetry of the cross section, the normal modes in a rectangular beam can be divided into four types [10], i.e.,

- Longitudinal *L*-modes: *u_x_* odd on *x* and even on *y*, *u_y_* even on *x* and odd on *y*, *u_z_* even on both *x* and *y*;
- Bending *B_x_*-modes: *u_x_* odd on both *x* and *y*, *u_y_* even on both *x* and *y*, *u_z_* even on *x* and odd on *y*;
- Bending *B_y_*-modes: *u_x_* even on both *x* and *y*, *u_y_* odd on both *x* and *y*, *u_z_* odd on *x* and even on *y*;
- Torsional *T*-modes: *u_x_* even on *x* and odd on *y*, *u_y_* odd on *x* and even on *y*, *u_z_* odd on both *x* and *y*.

Referring to [27], Lamé modes in a rectangular beam satisfy the following phase velocity condition:

(7) $$c_{p}=\sqrt{2}c_{T}$$

It is worth noting that the phase velocity condition of Lamé modes in a rectangular beam is consistent with that in an infinite plate [40]. Similarly, Lamé modes in a rectangular beam are formed by the reflection of shear waves at an angle of 45°, which exist only for discrete wavenumbers and frequencies. Especially, there are no Lamé modes for torsional modes, and the longitudinal and bending Lamé modes associated with height 2*b* and thickness 2*a* have wavenumbers given by

(8) $$\begin{array}{l} k_{L}=\left(2p-1\right)\frac{\pi }{2b},\;p=1,2,3\cdots \\ k_{L}=\left(2q-1\right)\frac{\pi }{2a},\;q=1,2,3\cdots \\ k_{B_{x}}=2m\frac{\pi }{2b},\;m=1,2,3\cdots \\ k_{B_{y}}=2n\frac{\pi }{2a},\;n=1,2,3\cdots \end{array}$$

Then, the frequencies of longitudinal and bending Lamé modes could be predicted according to their wavenumbers and phase velocities,

(9) $$\omega =k\cdot \sqrt{2}c_{T}$$

**Figure 3:**
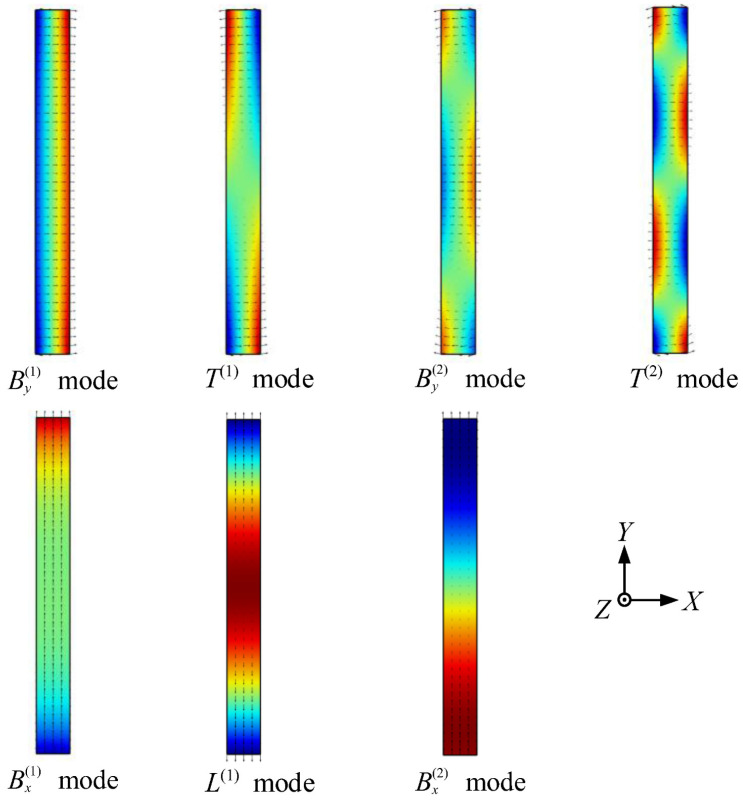
Mode shapes of 7 normal modes at 109.6 kHz, whereas *L*^(1)^ mode is the lowest Lamé mode. Arrow indicates the vector of in-plane displacement; color indicates the relative amplitude of out-of-plane displacement (blue: negative to red: positive).

The first Lamé mode with *k* = *π*/2*b* lies on the *L*^(1)^ branch, and the second Lamé mode with *k* = *π*/*b* lies on the $B_{x}^{\left(2\right)}$ branch, as shown in Figure 2. The displacement mode shapes of 7 normal modes, i.e., $B_{y}^{\left(1\right)}$, *T*^(1)^, $B_{y}^{\left(2\right)}$, *T*^(2)^, $B_{x}^{\left(1\right)}$, *L*^(1)^, and $B_{x}^{\left(2\right)}$ modes at the lowest Lamé frequency (109.6 kHz), are presented in Figure 3. The type of beam modes could be easily identified according to the symmetry of their respective displacement fields.

## 3. Guided Wave Scattering by a Through-Thickness Crack

### 3.1. Frequency Domain Analysis

The guided wave scattering in a rectangular beam with a through-thickness crack is modeled in COMSOL Multiphysics 5.6. As depicted in Figure 1, a cuboid with length *l* = 300 mm, thickness 2*a* = 2 mm and height 2*b* = 20 mm, is employed to represent the rectangular beam, with the absorbing region located at one end of the beam. The mass-proportional damping coefficient in the *z* direction varies following a cubic law [41]:

(10) $$\alpha =\{\begin{array}{c} 0,z∊[-l/2,l/2]\\ \alpha_{\max}{\left(\frac{|z|-l/2}{l_{\mathrm{abs}}}\right)}^{3},\;z∊\left(l/2,l/2+l_{\mathrm{abs}}\right] \end{array}$$

where the maximum damping coefficient *α*_max_ is 10 times the highest frequency to be investigated. The length of the absorbing region *l*_abs_ is 250 mm, larger than 3 times the longest wavelength in the waveguide, i.e., $B_{x}^{\left(2\right)}$ mode at the lowest frequency of investigation, which ensures the elimination of undesired boundary reflections.

To decouple the displacements of the two sides of the infinitely thin crack, a slit condition is introduced [42]. A typical crack with axial extent *a*_crack_ of 2 mm, width *w*_crack_ of 1 mm, and depth *d*_crack_ of 5 mm is given. The axial extent denotes the crack length in the axial direction, the sign of which depends on the crack orientation. The non-dimensional crack dimensions are introduced to simplify the problem. The relative crack width *W*%, is defined as a percentage of the beam thickness, given by *W*% = *w*_crack_/2*a* × 100%, while the relative crack depth *D*%, is defined as a percentage of the beam height, given by *D*% = *d*_crack_/2*b* × 100%. The geometry parameters and bulk wave velocities of the model are presented in Section 2. The incidence plane is located at *z* = −150 mm, while the reflection/transmission receiver planes are located at *z* = ±100 mm, each with 1000 uniformly distributed nodes. Referring to [41,43,44], the frequency-dependent displacement profile is input at the incidence plane to selectively generate the desired *L*^(1)^ mode. Subsequently, the extracted displacement and stress are used to determine the reflection and transmission coefficients at the receiver planes. According to the orthogonality and completeness of guided wave modes, an arbitrary wave field can be expressed as the linear superposition of various modes, with the amplitude *A_m_* of the *m*-th power-normalized mode given by

(11) $$A_{m}=-\frac{i\omega }{4}\int_{-b}^{b}\int_{-a}^{a}\sum_{j=x,y,z}(u_{j,m}^{*}\cdot \sigma_{jz,\mathrm{tot}}-u_{j,\mathrm{tot}}\cdot \sigma_{jz,m}^{*})dxdy$$

**Figure 4:**
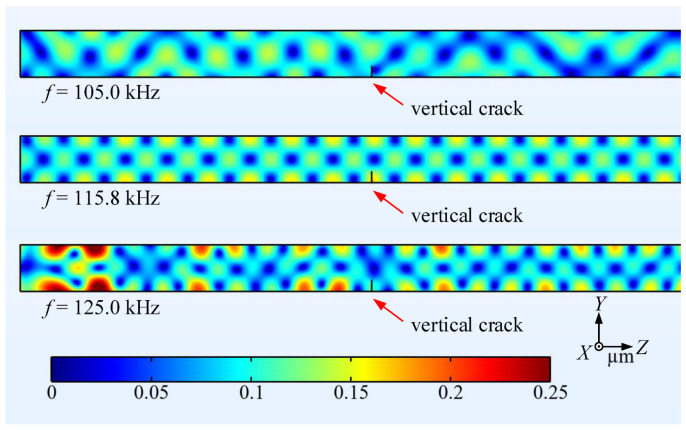
Surface displacement fields (magnitude) of the rectangular beam with a 25%-deep through-thickness vertical crack resulting from *L*^(1)^ mode incidence under various frequencies.

Harmonic analysis is carried out in the frequency domain to investigate the conversion behavior of *L*^(1)^ mode. The frequency ranges from 105 kHz to 125 kHz in steps of 0.2 kHz, which covers the lowest Lamé mode at 109.6 kHz. Using a stationary solver, the structural response is computed across all frequencies of interest in the beam. Figure 4 illustrates the surface displacement fields of the rectangular beam with a 25%-deep vertical crack resulting from *L*^(1)^ mode incidence at 105 kHz, 115.8 kHz and 125 kHz, respectively. It can be observed that the spatial periodicity along the *z* direction is destroyed, except in the second case at 115.8 kHz.

**Figure 5:**
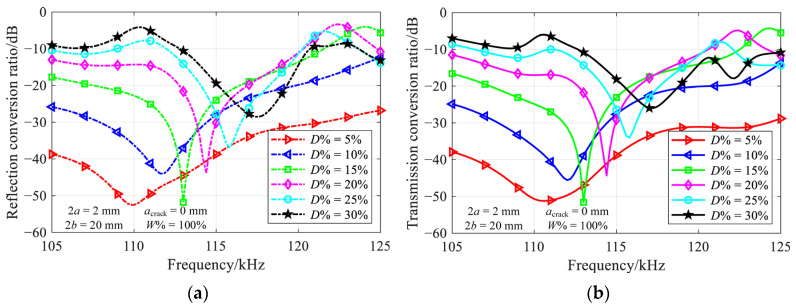
Variation of (**a**) reflection and (**b**) transmission conversion ratios with frequency for various depths of 2 mm-wide through-thickness vertical crack.

As non-propagating modes cannot transport energy by themselves [28], the conversion ratio at each receiver plane is defined as the energy ratio of converted propagating modes (excluding the forward propagating *L*^(1)^ mode) to all propagating modes:

(12) $$\begin{array}{l} C_{\mathrm{re}}=1-\frac{{\left|A_{L^{\left(1\right)}}\right|}^{2}}{\sum_{m=1}^{M}\left({\left|A_{-m}\right|}^{2}+{\left|A_{m}\right|}^{2}\right)},\mathrm{at}\;\mathrm{reflection}\;\mathrm{receiver}\;\mathrm{plane}\\ C_{\mathrm{tr}}=1-\frac{{\left|A_{L^{\left(1\right)}}\right|}^{2}}{\sum_{m=1}^{M}\left({\left|A_{-m}\right|}^{2}+{\left|A_{m}\right|}^{2}\right)},\mathrm{at}\;\mathrm{transmission}\;\mathrm{receiver}\;\mathrm{plane} \end{array}$$

where *M* is the number of propagating modes at a specific frequency, and the modes with the negative sign depict backward propagating modes. A larger conversion ratio indicates that more energy is converted to other propagating modes, resulting in a greater impact on the propagation of the incident mode induced by the crack. Figure 5 illustrates the conversion ratio for *L*^(1)^ mode incidence, where the relative crack depth ranges from 5% to 30% in increments of 5%.

In the case of a shallow vertical crack, the incident mode passes through the crack with weak mode conversion. As the vertical crack deepens, the frequency-dependent conversion ratio becomes complicated due to the significant mode conversion. Nonetheless, there is always a noticeable minimum at the same location in both reflection and transmission ratio diagrams. For instance, a minimum occurs at 115.8 kHz for a 25%-deep crack, which is consistent with the results presented in Figure 4.

### 3.2. Validation in Time Domain

To validate the results obtained from frequency domain analysis, ABAQUS/Explicit [45] is utilized to simulate the scattering of guided waves based on the same model shown in Figure 1. By placing overlapping duplicate nodes along a seam [46], a vertical crack of relative depth of 15% and 25% is introduced in the beam, respectively. A 35-cycle 115 kHz sine wave with a duration of 304 μs and bandwidth 20 kHz is selected as the excitation. The structure is discretized using eight-node brick elements with reduced integration (C3D8R). Since the shortest wavelength $\lambda_{\min}=2\pi /k_{B_{y}^{\left(1\right)}}\left(f_{\max}\right)\;=2\pi /554\times 1000\;\mathrm{mm}=11.34\;\mathrm{mm}$, the element size takes about 0.5 mm, which is smaller than one-twentieth of the shortest wavelength. The center mode shape technique, i.e., prescribing the displacement profile of the 115 kHz *L*^(1)^ mode at the beam end, is employed to excite the pure *L*^(1)^ mode.

**Figure 6:**
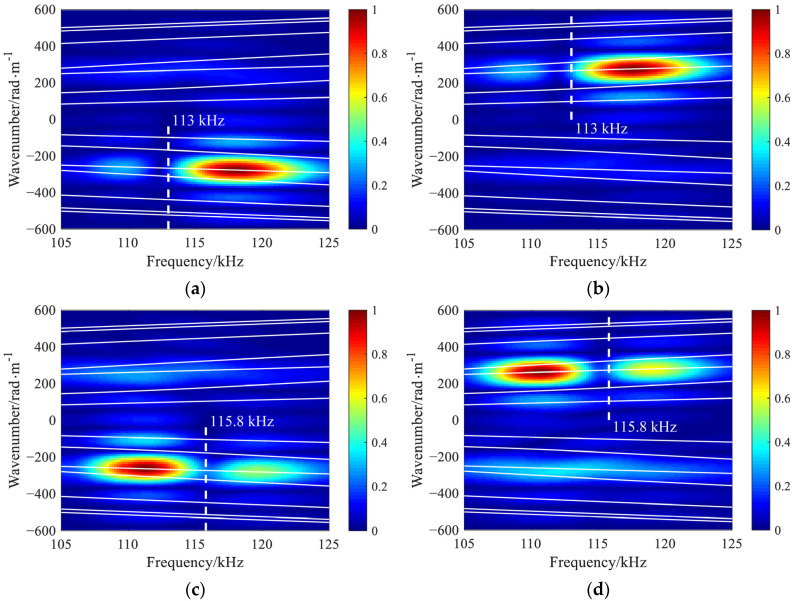
The reflected and transmitted frequency-wavenumber spectra for a through-thickness vertical crack with relative depth of (**a**,**b**) 15% and (**c**,**d**) 25% resulting from *L*^(1)^ mode incidence.

The presence of multiple modes in the reflected and transmitted signals due to mode conversion at the crack is observed, despite only one mode being excited. To analyze the mode conversion behavior, the two-dimensional Fourier transform is applied to reveal the energy distribution of various modes on the frequency-wavenumber spectrum. Two linear arrays with apertures of 60 mm, serving to receive reflected and transmitted signals, are located on both sides of the crack, 10 mm away from the origin. For the case of a shallow vertical crack, only the *y* component of the displacement on the beam surface is extracted since the forward propagating *L*^(1)^ mode still dominates in scattered signals, as shown in Figure 3. The frequency-wavenumber spectra of the transmitted and reflected signals are displayed in Figure 6.

A significant reduction in modal energy could be observed at a specific frequency in the reflected and transmitted frequency-wavenumber spectra, e.g., 113 kHz for 15%-deep crack, and 115.8 kHz for 25%-deep crack. These nearly perfect transmission frequencies are almost independent of modes, and could be expressed as a function of the crack depth. The slight deviations of the troughs in the transmitted frequency-wavenumber spectra may arise from the non-eliminated boundary reflections. Although both methods produce similar results, the frequency domain analysis is more efficient than the time domain simulation. Therefore, the frequency domain analysis is employed to calculate the scattering coefficients in Section 4.

## 4. Discussion

### 4.1. Effect of Crack Width

**Figure 7:**
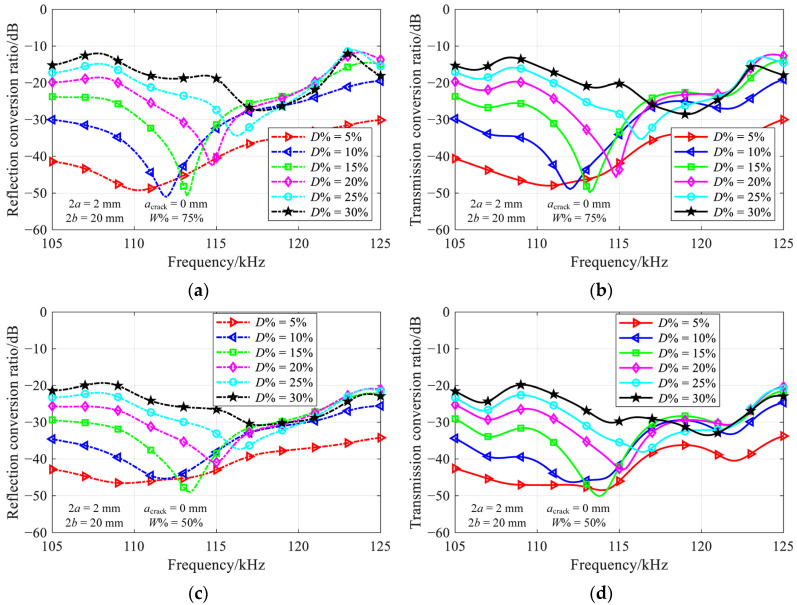
Variation of (**a**,**c**) reflection and (**b**,**d**) transmission conversion ratios with frequency in various depth-width combinations of part-through-thickness vertical crack.

Figure 7 shows the frequency-dependent conversion ratio for a part-through-thickness vertical crack, with the relative crack width of 75% and 50%, and the relative crack depth ranging from 5% to 30%.

Despite the variation of conversion ratios at the troughs, the nearly perfect transmission still occurs in most depth-width combinations. The non-ideal results for a relative crack depth of 5%, i.e., red lines in Figure 7, are attributed to the amplification of numerical errors in the case of weak mode conversion. In comparison to the results presented in Figure 5, a slight increase in the nearly perfect transmission frequency with a decrease of crack width could be observed. The results for relative crack width of 25% are not provided, due to the very weak mode conversion over the investigated frequency range.

### 4.2. Effect of Crack Axial Extent

**Figure 8:**
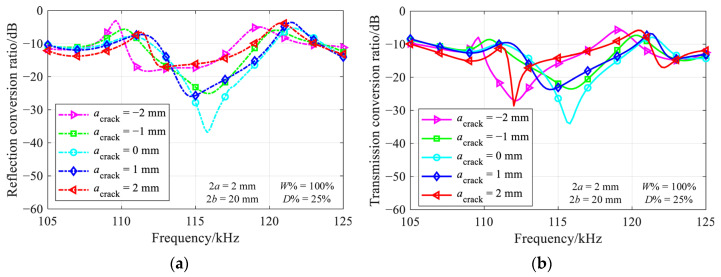
Variation of (**a**) reflection and (**b**) transmission conversion ratios with frequency for various axial extents of 25%-deep through-thickness inclined to crack.

The axial extent is an important parameter for an inclined crack. Figure 8 depicts the evolution of reflection and transmission conversion ratios for a 25%-deep through-thickness inclined crack with various axial extents.

The nearly perfect transmission phenomenon is not observed for inclined cracks, due to the absence of sharp troughs in the reflection and transmission conversion ratio diagrams. Besides, it should be noted that the mode conversion behavior for positive and negative axial extents is not symmetric about the zero axial extent, which differs from the results for the low-frequency SH0 plate mode, as reported in [6]. This discrepancy may be attributed to the greater number of modes present in the rectangular beam than in the infinite plate.

### 4.3. Effect of Beam Thickness

**Figure 9:**
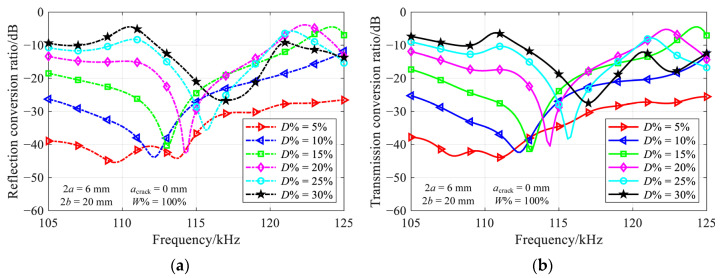

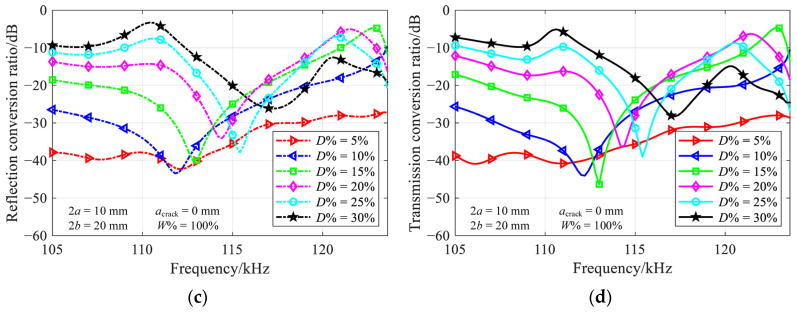
Variation of (**a**,**c**) reflection and (**b**,**d**) transmission conversion ratios with frequency in various depth-width combinations of through-thickness vertical crack.

To extend the applicability of our conclusions to the rectangular beam with various aspect ratios, two additional cases with beam height of 20 mm, beam thickness of 6 mm and 10 mm, are investigated. The evolution of conversion ratios resulting from *L*^(1)^ mode incidence for a through-thickness vertical crack in various depth-width combinations is displayed in Figure 9. Similarly, the nearly perfect transmission occurs in most depth-width combinations. Based on our results, the nearly perfect transmission frequency is barely related to the beam thickness.

**Figure 10:**
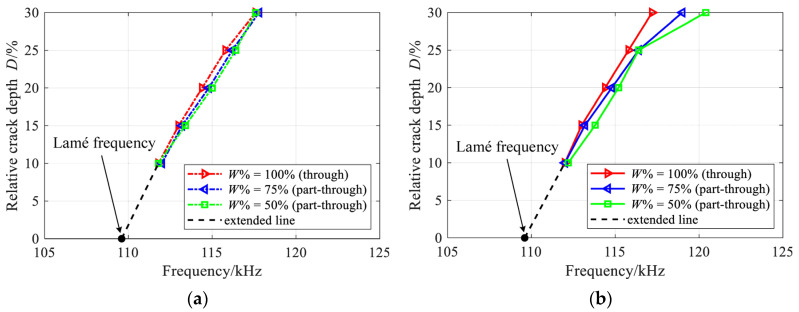
Comparison of frequencies with minimum conversion ratios at (**a**) reflection and (**b**) transmission receiver planes for part-through and through-thickness vertical cracks in a 2 mm-thick and 20 mm-high rectangular beams.

In Figure 10, the nearly perfect transmission frequency is presented for both part-through and through-thickness vertical cracks in a 2 mm-thick and 20 mm-high rectangular beams. It is obvious that, this frequency originates from the first Lamé frequency, which increases with the crack depth, but decreases with the crack width, both for the reflection and transmission. A more accurate frequency could be obtained if a smaller frequency step and finer mesh are adopted.

The low-frequency guided waves are crucial for field inspection, as they can propagate over long distances. Furthermore, the computational cost increases with the frequency dramatically due to the need for a finer mesh. Therefore, it is reasonable to focus on the nearly perfect transmission in the vicinity of the first Lamé frequency.

## 5. Conclusions

The interaction of the lowest Lamé mode with part-through and through-thickness cracks in the beam of the rectangular cross section is explored in this paper. The scattering coefficient of each reflected or transmitted mode is efficiently determined using the frequency domain analysis, and then verified through time domain simulation. Based on the numerical results, some conclusions are drawn:

1. The incident *L*^(1)^ mode will pass through the damaged region with negligible mode conversion if its frequency and the size of the part-through or through-thickness vertical crack satisfy a specific relation. However, such a nearly perfect transmission phenomenon is not observed for inclined cracks;
2. The nearly perfect transmission frequency originates from the first Lamé frequency, increases with the crack depth, whereas decreases with the crack width. Between them, the crack depth plays a major role in the frequency variation. Besides, the beam thickness has little influence on the nearly perfect transmission frequency;
3. The nearly perfect transmission frequency may serve as a potential indicator to evaluate the crack size in a rectangular beam. The results obtained in this study provide guidance for optimizing mode and frequency in the inspection;
4. The effect of the long-term phenomena of concrete on the dynamic behavior of prestressed rectangular beams has been studied experimentally and numerically through bending vibrations. In future work, the proposed numerical procedure will be combined with the prestressed analysis module available in commercial finite element software to investigate the mode conversion of guided waves at the crack along prestressed concrete beams.

## Data Availability

The main data supporting the findings of this study are available within the article. Extra data are available from the corresponding author upon reasonable request.
